# Risk factors for complications and poor function after open reduction and fixation of olecranon fractures

**DOI:** 10.1016/j.xrrt.2025.08.004

**Published:** 2025-08-18

**Authors:** Jawad Almasarweh, Hussam Ellauzi, Jan Dirk Theopold, Christian Kleber, Georg Osterhoff

**Affiliations:** a Department of Orthopaedics, Trauma and Plastic Surgery, University Hospital Leipzig, Leipzig, Germany; b Department of Trauma Surgery and Orthopaedics, BG Klinikum Unfallkrankenhaus Berlin, Germany

**Keywords:** Olecranon fracture, Plate osteosynthesis, Tension band wire, Elbow function, MEPS, OES

## Abstract

**Background:**

Surgical treatment of olecranon fractures is associated with a high rate of complications, potential restricted elbow function, and the need for revision surgeries. The aim of this study was to evaluate risk factors for complications and reduced functional outcomes following surgical treatment.

**Methods:**

All consecutive patients aged ≥18 years and treated at our level 1 trauma center for isolated open and closed olecranon fractures between January 2015 and March 2023 were retrospectively analyzed. Data on epidemiology, comorbidities, fracture type, treatment performed, and complications were collected. To assess elbow function, patients were contacted by phone or mail, and the Oxford Elbow Score, Modified Mayo Elbow Performance Score (MEPS), and Numeric Rating Scale of pain were recorded.

**Results:**

Fifty-two patients (age 47.9 ± 16.5 years, 56% male) were treated during the observation period. According to the AO Foundation/Orthopaedic Trauma Association classification, 61.5% of the cases were classified as type B and the remainder as type C fractures. 94.2% had closed fractures. Two Kirschner wire tension band was performed in 65.4% of patients, while the rest underwent locking plate fixation. The average functional scores were Oxford Elbow Score 42.6 ± 4.9, MEPS 80.1 ± 11.7, and Numeric Rating Scale 1.0 ± 1.5. Postoperative radiographs showed a mean intra-articular gap of 1.5 ± 2.2 mm (min./max. 0.0/11.0 mm) and an intra-articular step-off of 0.5 ± 0.6 mm (min./max. 0.0/2.2 mm). Thirty-one patients (59.6%) underwent a pure implant removal. In 8 patients (15.4%), a revision procedure was necessary, which went beyond just implant removal. These included reosteosynthesis for nonunion or inadequate reduction, arthrolysis, infection débridement, and unspecified revisions possibly related to new trauma. No significant differences were found regarding the need for secondary surgeries, implant removal, revisions, complications, or functional limitations with respect to fracture type, gender (*P* = .695), surgical procedure (*P* = .380), or postoperative joint gap/step-off (*P* = .462/.707). Patients who underwent a revision procedure beyond implant removal had worse function in the MEPS at follow-up (*P* = .011).

**Conclusion:**

Surgical treatment of olecranon fractures is associated with a high rate of complications, patients undergoing revisions beyond implant removal had poorer functional outcome; however, no significant differences in functional outcomes or secondary operations were found with respect to fracture type, gender, or surgical method. The size of the postoperative joint gap or step-off did not significantly affect the functional outcome. Despite acceptable functional scores, optimizing treatment and patient selection remains essential, as revisions are frequently necessary.

Olecranon fractures are common upper limb injuries, frequently occurring due to direct trauma or falls onto an outstretched hand. These fractures account for approximately 10% of all upper extremity fractures and are particularly prevalent among the elderly population due to osteoporotic bone structure and lower-energy trauma mechanisms.^34^^,^^37^ Treatment strategies range from conservative management to surgical intervention, with open reduction and internal fixation being the most commonly used approach for displaced fractures.^18^^,^^20^ However, surgical treatments carry the risk of complications across all age groups, which can range from infection, fixation failure, and postoperative elbow stiffness to symptomatic implant-related irritation.^22^ A lack of conclusive evidence has been highlighted in a recent systematic review, especially regarding the outcomes of various surgical options.^30^ Satisfactory outcomes in terms of preserved function were observed in selected low-demand geriatric patients, even in cases of widely displaced olecranon fractures with conservative management.^1^^,^^14^ In contrast, especially when it comes to displaced fractures, surgical management is typically preferred, either with plate fixation or tension band wire. Despite higher risks for complications like infection or symptomatic implants,^8^ plate fixation is often favored for more complex cases. Surgical complications are well-documented and include infections, which can occur in up to 8% of cases, nerve injuries in about 1%-4%, and osteosynthesis irritation^39^ in up to 15%. Additionally, nonunion or delayed union continues to be a concern, particularly in complex fractures,^5^ ranging up to 6%. Factors such as advanced age, comminuted fractures, and poor bone quality proved to increase the likelihood of complications and poor functional outcomes.^26^^,^^38^ In particular, older adults with osteoporotic bone are more prone to nonunion.^10^ Despite these insights, there is a need for more robust, high-quality data to better understand how these risk factors in elderly as well as other risk factors in younger populations affect long-term outcomes as well as the function of the elbow and how treatment protocols can be optimized for improved results.^29^ The aim of this study is to identify and correlate the significant risk factors for complications following olecranon fractures and their impact on postoperative functionality.

## Methods

A monocentric retrospective cohort study was conducted at our level 1 university trauma center. Through review of medical charts, the possible risk factors and complications were collected. All consecutive patients aged 18 years or older with isolated open or closed olecranon fractures who had undergone surgical treatment by either plate or tension band wire fixation in our hospital between January 2015 and March 2023 were enrolled and analyzed. Informed consent was obtained through telephone and recorded; written informed consent was obtained in cases where the data were collected through the questionnaire being sent by post.

**Figure 1:**
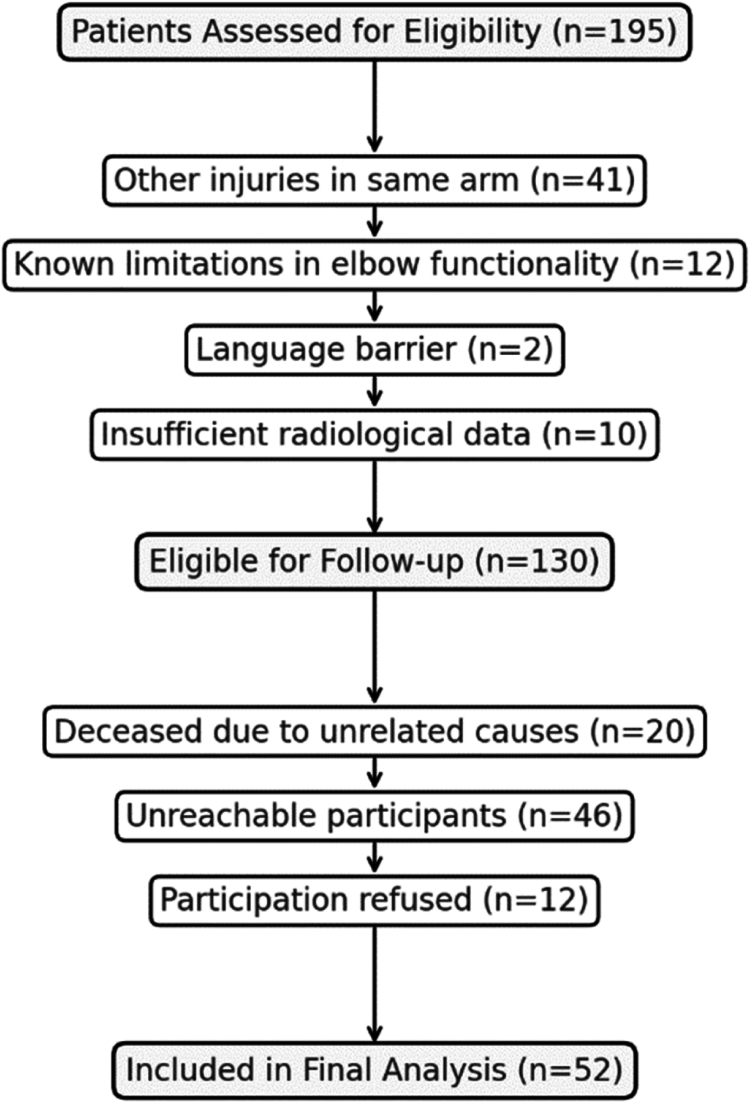
Patient enrollment Matplotlib.

Patients who had suffered additional ipsilateral arm injuries at the same time of the olecranon fracture, had a pervious known limitation of the functionality of the elbow, refused to take part in clinical research/studies, and who did not speak the German language were excluded (Fig. 1).

The study protocol had been approved by the local ethics committee (reference 109/24-ek).

Demographic data were collected from electronic medical records. This included gender, age, and type of trauma, surgical method, comorbidities, surgery time, length of hospital stay, and time to revision operation in days. The comorbidities included hypertension and diabetes mellitus and coagulation disorders. Perioperative radiological measurements of joint depression were obtained from radiographs and were included as risk factors. The data were transformed into table format for further processing after anonymization of personal data.

Complications such as implant-related irritation, infection, neurological deficits, and stiffness as well as hematoma and wound healing disorders were identified through patients' electronic records and our questionnaires, particularly in cases where these complications necessitated revision surgery. The preoperative and postoperative radiographs of the elbow were assessed by two surgeons at two different times. Postoperative intra-articular gap and step-off were measured in mm. The average of the two raters' measurements was used. Fracture complexity was assessed as well through the independent surgeons at separate times through the classification according to the AO Foundation/Orthopaedic Trauma Association classification.^21^ To ensure statistical robustness and minimize potential intrarater bias, only fracture types B and C were included in the analysis; further subclassification was not performed. Open and closed fractures were assessed and classified according to the Gustilo-Anderson system.^11^ Surgical factors were also considered, including the qualifications of the surgeon and the conditions under which the surgery was performed. Surgeon qualifications were classified according to the German medical training system as Assistenzarzt, Facharzt, and Oberarzt, corresponding to residents, attending, and consultants, respectively. Additional fellowship in trauma surgery was taken into consideration for consultants. The surgical conditions were divided into two groups: the first group represented routine, planned surgeries conducted between 8:00 am and 4:30 pm on weekdays, while the second group included unplanned surgeries occurring outside of regular working hours. Planned surgeries allow for optimal preparation and resources, while unplanned cases involve time pressure and reduced support, which might impact outcomes regardless of surgeon.

In order to evaluate the elbow functionality, three scores were obtained. The Oxford Elbow Score (OES), modified Mayo Elbow Performance Score (MEPS), and Numeric Rating Scale (NRS) for pain assessment. All included patients completed the functional scores at least one year after their initial surgery. The OES is effective with high reliability, validity, and responsiveness to changes in elbow function following treatment,^7^ as well as its German version, which was proved a suitable investigation tool for use in research settings.^19^ In addition, the Mayo Elbow Performance Score is an effective and reliable tool for evaluating elbow function.^25^ The standard Mayo Elbow Performance Score evaluates four categories: pain, motion, stability, and function. The German version of Mayo Elbow Performance Score (MEPS-G) was used, which typically lacks the assessment of elbow stability.^35^ Even though the NRS score focuses solely only on pain, it has been proved to be readily valid and can be used for treatment evaluation.^15^ Clear cutoff points for poor function have not been universally defined in the literature.^16^ However, lower scores on the OES and Mayo Elbow Performance Score (MEPS) are generally associated with greater disability. In this study, a cutoff point for poor function assessed through the OES was considered to be less than 35 points. This threshold is supported by existing literature and findings, particularly through the concepts of minimal important change and Patient-Acceptable Symptom State (PASS).^13^^,^^16^ Minimal important change refers to the smallest change in a score that patients perceive as meaningful, while PASS represents the point at which patients consider their condition acceptable. Together, these metrics provide valuable insights into the interpretation of patient-reported outcome measures, reinforcing the clinical relevance of using an OES score below 35 to identify poor function. The PASS for the MEPS score is typically considered to be 75 out of 100 following arthroplasty for primary elbow osteoarthritis; the modified MEPS version used has a maximum score of 90, where a direct proportional score of the PASS of the original MEPS would be 67. However, because patients with osteoarthritis often experience limited function, and to increase clinical relevance with a stricter evaluation of poor function, a poor score was defined as 61 instead.^17^ A score over three was defined to be a poor NRS.^36^

In this study, the term revision was used to refer to any secondary surgery going beyond simple implant removal, such as reosteosynthesis for nonunion or inadequate reduction, arthrolysis, infection débridement, and other unspecified revision surgeries, including those potentially related to new trauma. All specified revisions were performed at our institution over an 8-year period by different surgical teams, not necessarily by the same surgeon. The term secondary surgery used in this study included any surgical operation performed after the primary surgery, including implant removal. The differentiation between different types of surgeries was important because the frequency of implant removal which can occur up to 72%,^9^ thus significantly affecting the statistical power of other types of revisions. The data were then obtained through phone calls in which the informed consent was obtained and recorded. Patients who could not be reached after five phone calls made on different occasions were sent the same questions in a written questionnaire, accompanied by an informed consent form. Patients who did not complete the questionnaire within six weeks were considered nonresponders. Forty-six patients were not reached, and thus excluded from our analysis (Fig. 1). The gathered data were stored in an Excel database.

### Statistical analysis

All data were recorded in an Excel database (Microsoft Corp., Redmond, WA, USA) and exported to SPSS 29.0 (IBM Corp., Armonk, NY, USA) for statistical analysis.

Unless otherwise denoted, continuous data were summarized as mean ± standard deviation and categorical data as frequencies (n) and percentage (%).

Differences between surgical treatments were analyzed using nonparametric tests for continuous data and chi-square or Fisher's exact tests for categorical data. Inferential analyses were conducted to assess correlations. Correlation coefficients were categorized as follows: very weak (0-0.19), weak (0.2-0.39), moderate (0.4-0.59), strong (0.6-0.79), and very strong (0.8-1.0). The level of significance was defined as *P* < .05. Post hoc power analyses were conducted for key variables including intra-articular gap and step-off, fixation failure, fracture type, and their association with poor outcomes or complications. Eighty percent power and a significance level of 0.05 were used.

## Results

In total, 130 patients were initially considered eligible for the study. Of these, 20 patients died due to causes unrelated to the fracture, 12 patients declined to take part in the study, and 46 patients could not be reached for follow-up or participation. As a result, the final cohort of 52 patients was available for analysis (Fig. 1).

### Patients demographics

**Figure 2:**
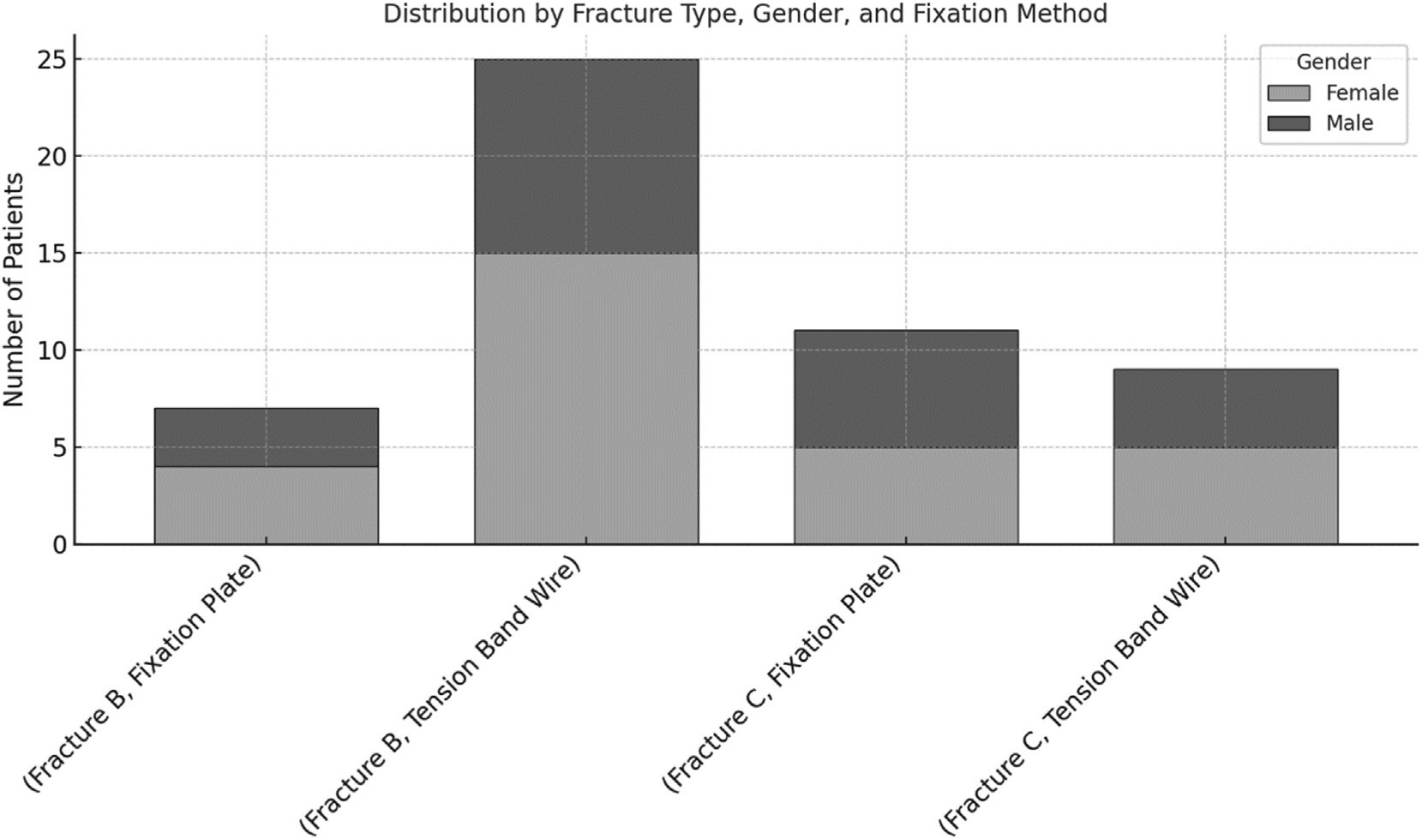
Patients distribution across fracture type, gender, and fixation method.

The patients' mean age was 47.9 ± 16.5 years (range of 21 to 83 years). Gender distribution was nearly balanced (55.8% male, 44.2% female). AO Foundation/Orthopaedic Trauma Association classification of the fractures revealed 61.5% of cases classified as type B and the remainder as type C. Three fractures were classified as Gustilo-Anderson Grade I open fractures, while the remaining 94.2% were closed fractures. Tension band wiring was performed in 65.4% of cases, 34.6% received angle stable plate osteosynthesis (Fig. 2).

Median length of hospitalization was 3 ± 3 days. The median preoperative joint depression was 0.0 ± 2.4 mm, with a maximum of 8.8 mm. Postoperative radiographic assessments revealed a median gap at the fracture site of 0.6 ± 1.5 mm ranging from 0.0 to 11.0 mm; the postoperative step-off had a median of 0.4 ± 0.8 mm and ranged from 0.0 to 2.5 mm.

### Surgical factors

**Figure 3:**
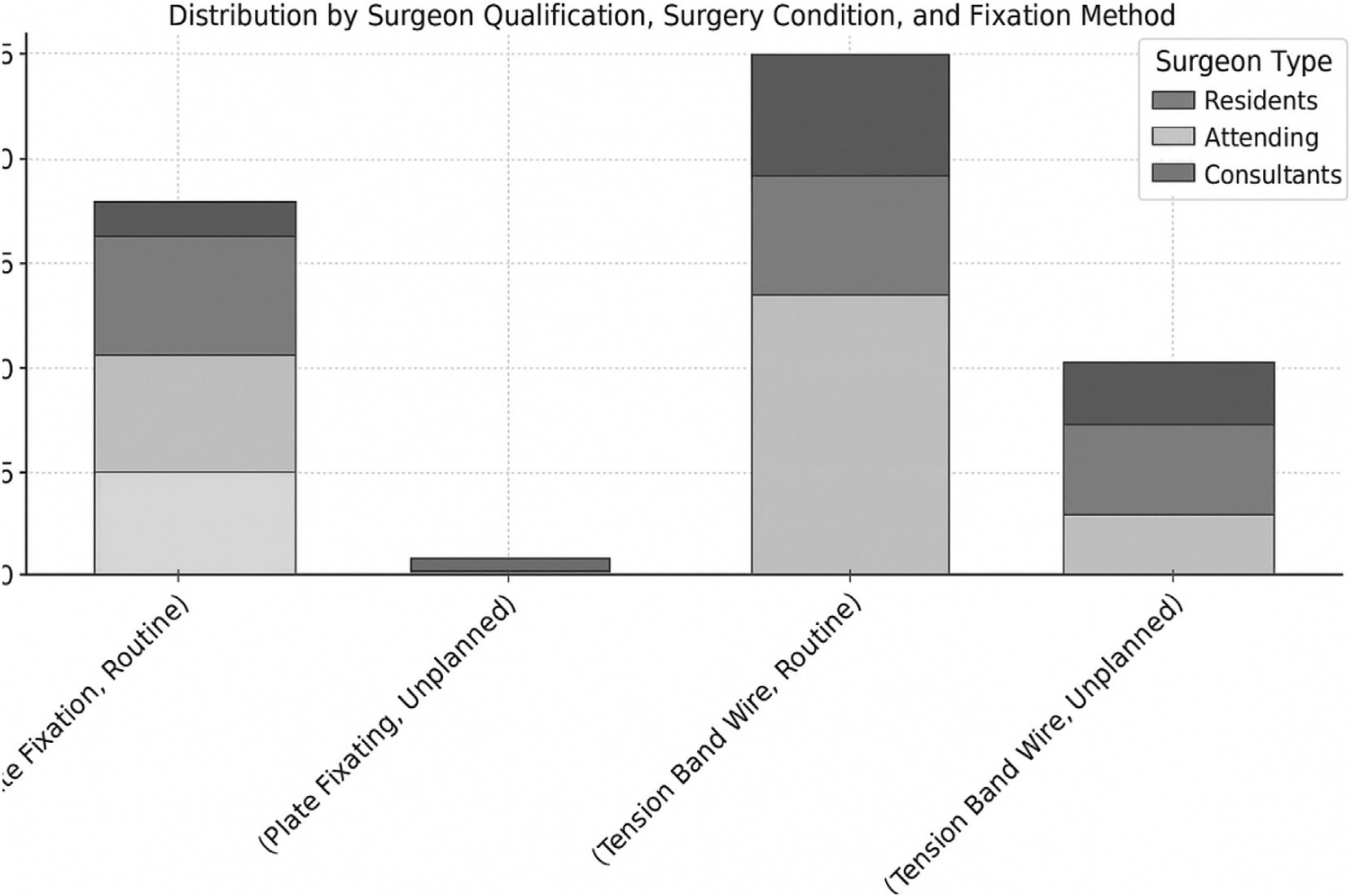
Data distribution across surgeon qualifications, surgery conditions, and fixation method.

A resident was the primary operator for 13 patients (25%), while an attending treated 17 patients (32.7%). The remaining patients were treated by consultants, all of whom had completed a fellowship in trauma surgery. Residents operated on four type C fractures (20%), attendings treated five (25%), and consultants handled the remaining 11 (55%). B fractures were almost equally distributed among the surgeon qualifications, with residents treating nine patients, Attendings 12, and consultants 11. Out of the cases treated by plate fixation, eight cases were carried out by consultants (44.4%), six cases (33.3%) by attending, and four cases through residents (22.2%). The distribution of tension band wire revealed nine cases (26.5%) performed by residents, 11 (32.4%) by attending, and the rest (42.2%) by consultants (Fig. 3).

Forty-two surgeries (80%) were planned, while the remaining 10 surgeries (19.2%) were unplanned. Residents and attending each performed 24 routine planned surgeries equally distributed, while the remaining 18 surgeries (42.9%) were unplanned. Most unplanned surgeries involved tension band wire fixation (90%) and were mainly operated through attending (50%) and consultants (40%). Fracture classification distribution in the unplanned surgeries was nearly balanced (60% B, 40% C).

### Secondary surgery

Thirty-nine patients underwent a secondary operation (75%). Secondary surgeries included implant removal as well as revisions. Median time to a secondary surgery was 367 ± 293 days, with substantial variability among patients. Seventeen patients (43.6%) suffered from fracture type C. Using Spearman correlation to determine the association between studied variables and the occurrence of secondary surgeries. Variables like age and fracture type were expected to be associated with higher overall secondary surgery rates, but this was not significantly supported by the data (*P* = .967/.195). Additionally, factors such as surgery duration, gender, injury type, length of hospital stay, open fractures, and the presence of comorbidities like hypertension, coagulopathies, and diabetes did not show a significant correlation with the likelihood of a secondary surgery. Furthermore, surgical factors including the qualification of the surgeon or surgical conditions did significantly correlate with the risk of secondary surgery. Unexpectedly, the presence of more than 2 mm intra-aticular gap or step-off did not significantly correlate with the occurrence of a secondary surgery (*P* = 1.000/.569).

### Implant removal

Thirty-one patients underwent a pure implant removal (59.6%). Further analysis revealed that 20 (64.5%) of these patients had a B fracture. The fixation methods were also varied, with 22 patients treated with tension band wiring and the remaining patients treated with plate fixation (29%). Median time to implant removal was 401 ± 315 with high variability. Spearman correlation to determine the association between studied variables and the occurrence of implant removal. Factors such as age and gender did not significantly correlate with the increased rate of implant removal (*P* = .890/.340). Furthermore, the expected correlation between fracture type or fixation method could not be significantly proven (*P* = .965/.313). Additionally, the presence of comorbidities like hypertension, coagulopathies, and diabetes did not show a significant correlation with the need for implant removal. Surgical factors and surgical conditions did not have any significant impact on the occurrence of implant removal. Open fractures did not significantly correlate with the incidence of implant removal (*P* = .148). There was no significant correlation between implant removal and the presence of more than 2 mm intra-aticular gap or step-off (*P* = .860/.416).

### Revision

In eight patients (15.4%), a revision procedure was necessary, which went beyond just implant removal, where four patients. (50%) underwent reosteosynthesis, 2 patients (25%) required arthrolysis. One patient (12.5%) underwent débridement for infection, and one had an unspecified revision, possibly trauma-related. Three patients had B fracture, while the rest (62.5%) suffered a C fracture. All patients undergoing revisions had closed fractures. These distributions between types of fixation were exactly equal in these patients. Median time to revision was 63 ± 81 days. Using Spearman correlation to assess the association, variables such as fracture type or fixation method did not significantly correlate with the occurrence of revision (*P* = .134/.330). Furthermore, factors like age and gender as well as presence of comorbidities did not significantly correlate with the risk of revision. Additionally, the surgical factors including qualification of the surgeon or surgical conditions did not significantly correlate with the occurrence of revisions. The findings revealed no significant correlation between the presence of 2 mm or more intra-aticular gap or step-off with the occurrence of a revision surgery (*P* = .810/.674).

### Functional outcomes

Clinical outcome measures showed a median OES of 43 ± 6, a median MEPS (Mayo Elbow Performance Score) of 87.5 ± 15, and a median NRS (Numerical Rating Scale for pain) of 0 ± 2. Patients were categorized into 2 groups for each score. The poor OES defined as being lower than 35 points, poor Modified Mayo Elbow Performance score (MEPS) as being lower than 61 points, and poor NRS being more than 3 points. Using Spearman correlation, a strong significant correlation was observed between poor Modified Mayo Elbow Performance score (MEPS) and poor NRS (r = 0.627, *P* < .001), highlighting that higher pain levels were consistently accompanied by poorer functional performance. Modified Mayo Elbow Performance score (MEPS) and OESs exhibited a weak positive correlation (r = 0.386, *P* = .005) demonstrating that these two measures aligned in their assessment of postoperative function.

Spearman correlation was used to access the correlation between studied variables and poor functional outcomes. Open fractures had more pain as indicated by low NRS score (*P* = .035), however with no significant correlation with poor OES or MEPS (*P* = .666/.308). Variables such as age, gender, or fixation method did not significantly correlate with poor functions.

Patients who underwent surgery performed by an attending exhibited a weak correlation with poor functional outcomes, as indicated by low MEPS and NRS scores (*P* = .018/.010). However, this correlation was not observed when the surgeon was either a resident or a consultant. Regarding surgical conditions, the planned routine operation also demonstrated a weak correlation with poor MEPS and NRS scores (*P* = .006/.032). Mann-Whitney U did as well confirm the correlation between surgery performed by an attending or planned routine operation with poor MEPS score (*P* = .02/.007).

Even though the complexity of fracture was expected to have poorer function, this was not significantly proved (OES, *P* = .854, MEPS, 0.802). The presence of comorbidities, including diabetes mellitus, did not show significant correlation with poorer functions. The distribution of radiographic parameters such as postoperative intra-articular step-off and gap was tested using the Mann-Whitney *U* test; these were not associated with poorer functional outcomes (OES, *P* = .974/*P* = .451, MEPS, *P* = .410/*P* = .230, NRS, *P* = .410/*P* = .451). Furthermore, the Mann-Whitney *U* test showed no significant association between fracture type and poor functional outcomes (OES, *P* = .832, MEPS, *P* = .256), where the fracture type was tested against poor function as defined in this study. Chi-square tests showed as well no significant association between gender and poor outcome measures such as Oxford scores (χ^2^ = 0.153, *P* = .695) and MEPS (χ^2^ = 0.006, *P* = .937).

Moreover, Spearman correlation showed no statistically significant relationship between the occurrence of a secondary surgery and poor functional scores (OES, *P* = .737, MEPS *P* = .819). The lack of significant correlation with poor functional score was also observed in patients who underwent an implant removal (OES *P* = .349, MEPS *P* = .074). Patients who underwent a revision showed a weak correlation with a poorer functional outcome as demonstrated by poor MEPS (r = 0.300, *P* = .031) and poor NRS (r = 0.352, *P* = .011). Patients who had revision surgery demonstrated lower scores in the MEPS at follow-up (*P* = .011).

### Postoperative complications

None of the studied variables, including age or the presence of comorbidities like hypertension, showed a significant correlation with the occurrence of postoperative complications (*P* = .774/.105). However, once the complications were categorized into stiffness, neurological complications, fixation failure, malunion/nonunion as well as wound healing disorders, a weak correlation was established between a fracture type and fixation failure (r = 0.363, *P* = .009), but without significant correlation with any of the surgical factors. Furthermore, there was no significant correlation but a trend between the fracture type and the occurrence of postoperative complications (*P* = .062). The presence of postoperative complication tended to have a moderate correlation with the occurrence of implant removal (*P* = .003) and a strong correlation with revision risk (r = 0.852, *P* = < .001). No significant correlation was found between complications and open fractures (*P* = .457).

## Discussion

The aim of this study was to identify and correlate significant risk factors for complications following isolated olecranon fractures and their impact on postoperative functionality. We found a weak correlation between fixation failure and fracture type and saw poorer function after revision surgery for reasons other than purely implant-related irritation tended.

Although postoperative complications were common, revision surgeries and radiographic measures, such as intra-articular step-off or gap, did not show a statistically significant relationship with functional measures.

A review of the existing literature reveals that the association between revisions beyond simple implant removal and poorer functional outcomes is a novel finding and was not previously reported. Buijze G et al^4^ observed an improvement in the range of motion following implant removal in patients treated with plate fixation, suggesting that soft tissue irritation and adhesions limit function. Previous studies have reported that more complex fractures, such as Monteggia injuries, are associated with higher reoperation rates.^3^ While these findings may not be directly applicable, they can be transferred to our results, which also show a weak correlation between fracture type and fixation failure in type C fractures. However, our study did not identify a significant association between fracture type and the overall risk of secondary surgeries, revisions as well as implant removal.

We found a weak correlation between the primary surgeon being an attending as well as routine operation and poor functional outcomes. Due to the small subgroups, adjusting for potential confounders like fracture pattern and patient age was not possible. Hence, this correlation should be regarded as what it is: an association, not necessarily a causal relation.

Ozdag et al^24^ evaluated the outcomes of procedures performed by the same surgeon before and after completing fellowship training. Despite the increase in experience and specialization, no significant differences in functional outcomes were observed, suggesting that surgical experience alone may not be a determining factor in patient-reported outcomes. However, this study was conducted with a surgeon who had completed fellowship training, which limits the direct applicability of these findings to our current context. The observed increase in complications in planned surgeries may be attributed to several factors, including the relatively small overall sample size. Additionally, the limited sample size (n = 10) in unplanned surgeries further complicates the ability to make generalizable conclusions about the relationship between surgical condition and complication rates, thus limiting the statistical power to draw definitive conclusions.

Our study found no significant correlation between age or gender and rate of secondary surgeries, implant removal, or even revision rates. This finding did not well aligned with the current literature,^6^ which described a higher rate for secondary surgeries, mainly due to implant removal in younger population and in women. The differentiation between secondary surgery and revision was not described, and it is worth mentioning that the secondary surgeries in that case were mainly requested and not necessary due to a complication.

Another study showed the revisions are higher in older populations and mainly due to osteoporotic bone.^33^ However, our results aligned with more recent studies, which showed no significant correlation between age, gender, or diabetes and postoperative complication rate in olecranon fractures.^15^^,^^32^ Furthermore, our findings regarding fracture classification and its lack of significant association with functional outcomes as well as revision operations align with current literature.^2^^,^^31^ The weak correlation observed between fixation failure and fracture type did not align with previous literature,^31^ which found that specific fracture patterns did not influence fixation stability. However, the limited number of fixation failures suggests that, while there is some indication of a relationship between fracture type and fixation failure, the low occurrence of this specific complication may have influenced the statistical power of this finding. In our study, the relationship between fracture type and the occurrence of postoperative complications was not found to be statistically significant. This may primarily be attributed to the limited number of cases and the use of various fixation methods, where no clear correlation to an increased risk of complications. These findings contrast with the current literature, which reports a higher incidence of complications associated with tension band wiring.^22^

A systematic review of 1,518 patients reported that a loss of reduction was associated with an intra-articular joint gap or step-off greater than 2 mm.^30^ The review indicated that loss of reduction was more common with tension band wiring; however, functional outcomes, as measured by the Mayo Elbow Performance Score (MEPS), still showed good to excellent results. Furthermore, research showed that a better radiological anatomical reduction can be achieved with plate fixation in comparison to tension band wiring, although there was no significant difference in function, measured in elbow range of motion.^12^ These findings align with our own observations, where the size of the postoperative intra-articular joint gap or step-off did not significantly affect functional outcomes. Putnam et al^28^ showed good to excellent functional outcomes with nonoperative treatment of simple olecranon fractures among all adults' age groups. Conservative management is often characterized in the literature by a maximum displacement of 2 mm.^23^ These findings suggest that full anatomical reduction may not significantly impact functional outcomes, which aligns with our own results. It remains crucial to define the acceptable range for anatomical reduction of the articular surface, which, when not met, can lead to postoperative stiffness and secondary osteoarthritis^27^ ultimately leading to decreased functional outcomes.

Clinically, this study underscores the importance of tailoring treatment strategies to individual patient characteristics, particularly focusing on minimizing postoperative complications and optimizing pain management to improve outcomes. The clinical relevance of fixation failures remains unclear, particularly in complex fracture patterns where functional outcomes do not always correspond with radiographic findings or clear risk factors. In light of this knowledge gap, and given the observed rates of complications and revisions, future research should include multicenter, prospective studies with larger cohorts. These studies should stratify the outcomes of fracture complexity and failure mode and include long-term follow-up to evaluate the influence of fixation failure on complications, functional recovery, and the need for secondary procedures. Incorporating standardized radiographic evaluation and patient-reported outcome measures will help to identify which fixation failures are clinically significant and will assist in the development of clearer revision criteria. Additionally, prospective studies assessing both preoperative and postoperative function should investigate the role of implant removal to better determine its clinical relevance, optimal indications, and timing.

### Limitations

Several limitations could be considered. The small sample size of 52 patients and the heterogeneity of the study population reduce the statistical power to detect smaller effects, may limit the generalizability of the findings, and may have introduced variability in the outcomes. Post hoc power analyses indicated that future studies would require between 80 and 260 patients, depending on the studied variable, to enhance the robustness of the statistical analyses. Furthermore, the absence of formal intrarater and inter-rater reliability analysis for the radiographic assessments may affect the reproducibility and objectivity of the measurement process. The retrospective design also carries inherent biases, such as selection bias and incomplete data collection. An important limitation is the lack of assessment for the presence or degree of extension deficit, which could have provided additional insight into functional recovery. Moreover, functional outcomes were only evaluated before or after revision surgery, which limits the ability to fully assess the impact of revision procedures on recovery in the same patient.

## Conclusion

This retrospective case series found that while fixation failure occurred more frequently in complex olecranon fractures, it did not significantly affect functional outcomes. The size of the intra-articular gap or step-off, as assessed on standardized postoperative radiographs, showed no association with functional results or revision rates. No significant differences were observed based on fracture type, patient demographics, or surgical technique.

## Acknowledgment

The authors would like to thank the participants for their cooperation and their trust in the authors’ research.

## Disclaimers:

Funding: No funding was disclosed by the authors.

Conflicts of interest: The authors, their immediate families, and any research foundations with which they are affiliated have not received any financial payments or other benefits from any commercial entity related to the subject of this article.
